# Cortical midline involvement in autobiographical memory

**DOI:** 10.1016/j.neuroimage.2008.09.033

**Published:** 2009-02-01

**Authors:** Jennifer J. Summerfield, Demis Hassabis, Eleanor A. Maguire

**Affiliations:** Wellcome Trust Centre for Neuroimaging, Institute of Neurology, University College London, 12 Queen Square, London WC1N 3BG, UK

**Keywords:** fMRI, Episodic memory, Retrieval, Imagination, Self, Reality, Medial prefrontal, Retrosplenial, Posterior cingulate

## Abstract

Recollecting autobiographical memories of personal past experiences is an integral part of our everyday lives and relies on a distributed set of brain regions. Their occurrence externally in the real world (‘realness’) and their self-relevance (‘selfness’) are two defining features of these autobiographical events. Distinguishing between personally experienced events and those that happened to other individuals, and between events that really occurred and those that were mere figments of the imagination, is clearly advantageous, yet the respective neural correlates remain unclear. Here we experimentally manipulated and dissociated realness and selfness during fMRI using a novel paradigm where participants recalled self (autobiographical) and non-self (from a movie or television news clips) events that were either real or previously imagined. Distinct sub-regions within dorsal and ventral medial prefrontal cortex, retrosplenial cortex and along the parieto-occipital sulcus preferentially coded for events (real or imagined) involving the self. By contrast, recollection of autobiographical events that really happened in the external world activated different areas within ventromedial prefrontal cortex and posterior cingulate cortex. In addition, recall of externally experienced real events (self or non-self) was associated with increased activity in areas of dorsomedial prefrontal cortex and posterior cingulate cortex. Taken together our results permitted a functional deconstruction of anterior (medial prefrontal) and posterior (retrosplenial cortex, posterior cingulate cortex, precuneus) cortical midline regions widely associated with autobiographical memory but whose roles have hitherto been poorly understood.

## Introduction

Recollecting autobiographical memories of personal past experiences is an integral part of our everyday lives and relies on a distributed set of brain regions that includes the hippocampus, parahippocampal gyrus, lateral temporal cortices, temporo-parietal junction, lateral prefrontal cortex, thalamus, and cerebellum (Cabeza and St Jacques, 2007; Maguire, 2001a; Maguire and Frith, 2003a; Svoboda et al., 2006). Classically, neuroimaging studies of autobiographical memory have focused on characterising the role of the hippocampus during retrieval (e.g. Addis et al., 2004; Gilboa et al., 2004; Maguire and Frith, 2003b; Ryan et al., 2001). However, these studies also reveal large spatially-extended swathes of activity across cortical midline structures encompassing posteriorly the retrosplenial cortex (RSC), posterior cingulate cortex (PCC), and precuneus, and anteriorly the dorsal and ventral medial prefrontal cortex (mPFC) (Buckner and Carroll, 2007; Hassabis et al., 2007a; Schacter et al., 2007). Surprisingly little is understood about the functional contributions these midline brain regions make to the overall recollective experience. Progress has been hindered not only by limited neuropsychological evidence, where focal lesions in midline regions are rare (Bird et al., 2004; Maguire, 2001b), but also by the tendency to consider these regions, especially within posterior portions of the brain, as a single functional unit. It is unlikely, however, that a unitary function is being performed either posteriorly or anteriorly, particularly as the activations subsume cytoarchitecturally distinct regions (Cavanna and Trimble, 2006; Ongur et al., 2003; Saleem et al., 2008; Vogt and Laureys, 2005; Vogt et al., 2006).

The ubiquity and prominence of these cortical midline activations during neuroimaging suggest that functional deconstruction of these extended brain areas is critical to understanding the processes and mechanisms underpinning our ability to re-experience the past, and may throw new light on other cognitive functions such as imagination, thinking about the future and spatial navigation that also engage some of these brain areas (Bar, 2007; Buckner and Carroll, 2007; Hassabis and Maguire, 2007; Schacter and Addis, 2007). In the current study we considered two defining features of autobiographical memory that may offer some clues about functional subdivisions within these midline cortical regions: their self relevance (‘selfness’; by this we mean events that happen to, and so are personally experienced by, the self), and the fact that they occur externally in the real world (‘realness’; in contrast to those events created and played out internally in one's imagination).

Autobiographical memories are personal and highly self-relevant, tightly bound to an individual's self-schema (Conway and Pleydell-Pearce, 2000; Tulving, 1983, 2002). Previous neuroimaging studies have contrasted autobiographical memory retrieval with a range of other tasks that were less self-relevant such as rest, semantic memory retrieval or the exposure to other people's autobiographical memories, and found cortical midline regions were more active for autobiographical memory (for a recent meta-analysis see Svoboda et al., 2006). Surprisingly, although several studies have included both autobiographical and non-autobiographical/non-self conditions, few have directly discussed this comparison in terms of selfness (e.g. Szpunar et al., 2007; Maguire and Frith 2003a). Instead, evidence from other types of self processing has typically been called upon in the interpretation of activations in autobiographical memory studies. Manipulations involving the retrieval and judgement of self-relevant personality traits (D'Argembeau et al., 2007; Johnson et al., 2002; Kelley et al., 2002; Kjaer et al., 2002; Lou et al., 2004; Macrae et al., 2004; Moran et al., 2006), differences in viewpoint perspective (e.g. first or third person) (D'Argembeau et al., 2007; Ruby and Decety, 2001; Vogeley and Fink, 2003; Vogeley et al., 2004), and mentalizing about the thoughts or actions of oneself or others (Frith and Frith, 1999, 2003; Gallagher and Frith, 2003; Mitchell et al., 2006; Saxe, 2006), commonly result in activation of midline structures and in particular portions of the mPFC and PCC (e.g. see Amodio and Frith, 2006; Gillihan and Farah, 2005; Northoff et al., 2006; Schmitz and Johnson, 2007 for reviews). Thus it has been suggested that activation of mPFC and PCC during autobiographical memory may be linked to self-referential processing (Cabeza and St Jacques, 2007; Hassabis et al., 2007a; Svoboda et al., 2006). Rather than making inferences from self processing in other domains, in the current study we focused on delineating the brain areas modulated by self processing specifically associated with autobiographical memory.

Another key feature of autobiographical events is their realness, they occur in the external world. Little is known, however, about how the brain distinguishes real from fictitious experiences, although two recent studies suggest that cortical midline areas may also play a role in supporting this crucial ability (Abraham et al., 2008; Hassabis et al., 2007a). Abraham et al. (2008) had participants make judgements about scenarios involving famous people or fictional characters. The authors found that events involving famous people preferentially engaged anterior mPFC and PCC/precuneus, although differences in associated detail, familiarity and greater self-relevance/social similarities with real people may have influenced the results. Hassabis et al. (2007a) adopted a different approach by directly comparing recall of recent real autobiographical “snapshot” memories (scenes) with recall of previously imagined fictitious scenes, matched for the amount of detail and vividness. While both types of scenes activated several areas of the memory network in common, PCC, precuneus and anterior mPFC were preferentially engaged when real autobiographical scenes were retrieved. However, in the Hassabis et al. (2007a) study real autobiographical scenes were both real, *and* more self relevant compared with imagined scenes. Consequently, this study could not distinguish between activations representing selfness and those representing the realness aspects of the memories.

The aim of the current study, therefore, was to systematically investigate how selfness and realness modulate cortical midline structures during the retrieval of autobiographical experiences. In order to achieve this, memory for recent real autobiographical events was compared with memory for recent events involving other individuals and from which the participant was absent, but which were nevertheless similarly rich, detailed, complex, and naturalistic. These non-self events were of two types, derived from viewing a feature length movie (“Sideways”; Twentieth Century Fox, Fox Searchlight Pictures, Beverly Hills, California; 2004) or from television (TV) news clips. There is a relative dearth of studies in the literature that have used extended movies as stimuli in memory experiments, although the use of such stimuli has been suggested as an effective way to study naturalistic cognitive processing (Furman et al., 2007; Hasson et al., 2008; Spiers and Maguire, 2007). In addition, participants recalled fictitious (plausible) events previously created internally in their imagination. Imagined autobiographical (self) events included the active involvement of the participant (rather than static scenes employed by Hassabis et al., 2007a). Imagined film events were plausible events that could have happened (but didn't) to the characters in the film, while imagined news events were plausible events that never actually happened but that could have been reported on a UK TV news programme.

This experimental design thus allowed us to dissociate the selfness and realness of complex events in a novel way not possible in previous studies, providing much-needed insights into the neural basis of these critical features of autobiographical memory. We hypothesised that our manipulations would result in differential modulation of cortical midline structures both anteriorly and posteriorly, and so would facilitate the functional deconstruction within these brain areas. The cortical midline structures of specific interest therefore included dorsal and ventral medial prefrontal cortex, anterior cingulate cortex, precuneus, posterior cingulate cortex, retrosplenial cortex, and the thalamus.

## Methods

### Participants

Eighteen healthy right handed native English speakers took part in the study (11 females; mean age 25.1 years, SD 3.7). Only participants who had not previously viewed the film “Sideways” and were naive to its content were included. All participants had normal or corrected to normal vision and gave informed written consent in accordance with the local research ethics committee.

### Task and procedure

**Fig. 1:**
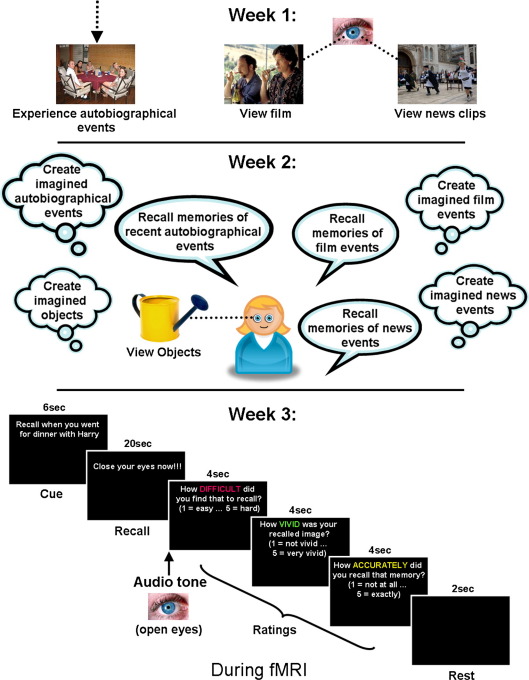
Schematic of the experimental structure over three consecutive weeks. Week 1: Participants experienced autobiographical events in their own lives. In addition they watched the feature-length movie “Sideways”, and watched a selection of short clips featuring minor news events. Week 2: One week later participants returned and their memory for recent autobiographical events as well as events from the movie and news clips was tested. Participants then created imagined autobiographical scenarios, imagined events involving the characters of the film and imagined news events. They also viewed a selection of acontextual objects on a computer screen and created images of acontextual objects in their imagination. Week 3: A memory recall task was performed during fMRI. Trials began with a text cue informing participants which real or previously imagined event/object to recall (further examples of cues are given in Table 2), followed by 20 s during which they re-experienced that event/object with their eyes closed. After an audio tone sounded, participants opened their eyes and performed a series of ratings (see Methods).

The experiment comprised three sessions conducted over three consecutive weeks (see Fig. 1): In week 1 (*viewing* session), participants experienced the normal everyday events in their lives and viewed the movie and the news clips. In week 2 (*interview* session) they recalled autobiographical experiences, events from the movie and the news events. In addition, they created events of each category in their imagination. In the third week (*MRI scan* session) they were scanned using fMRI during which they recalled the real and imagined events. For each real and imagined event condition there was also a control task where participants recalled previously viewed (real) or previously imagined acontextual single objects respectively, thus controlling for basic recall and visualisation effects. All viewings, interviews and behavioural tests were conducted by the same experimenter in the same testing room on the same day (and time) of successive weeks.

#### Viewing session (week 1)

The first session exposed participants to naturalistic events that would be used as non-self stimuli and were informed they would be required to remember the events in later sessions. Participants viewed the movie “Sideways” (duration 2 h) on a computer monitor (PowerDVD, Cyberlink). Sideways was selected for this experiment after extensive consideration of a broad range of movies. Sideways involves a relatively simple plot, and the events that occur are relatively comparable to those that could occur in real life. After a break, participants then viewed 12–17 recent news clips (average number 12.6 clips) selected from internet news podcasts (BBC London http://news.bbc.co.uk/1/hi/programmes/4977678.stm; Sky news http://news.sky.com/skynews/podcasts). Edited news clips (1–2 min in length; mean duration 1 min 20s; MPEG Streamclip 1.1, Squared 5; MEncoder Windows Gui, Hans-Carl Overdalhoff) were played on a computer monitor (Windows media player, Microsoft Corporation) and contained reports of minor news events (emotionally neutral or slightly positive) from around the world that had occurred within the four weeks prior to each participant's first session. News clips that participants had previously seen or heard, had personal experience of, or that elicited specific memories were excluded from future sessions.

#### Interview session (week 2)

One week later, participants returned for an interview session. There were eight different tasks. Firstly, recent autobiographical memories from the previous four weeks were described out loud by the participant (RS; real self events). The ten memories selected for inclusion for each participant were vivid, specific in time and place, with a relatively circumscribed time frame, and emotionally neutral or slightly positive in nature. Secondly, memories of experimenter-selected events from the film Sideways were assessed (RF; real non-self film events). Events were memorable, relatively neutral in emotional content, comparable to real everyday events and involved the main film characters. Participants described out loud details of each event to confirm they remembered it accurately and ten very clearly remembered memories of film events were selected for inclusion. Events that elicited strong personal memories were excluded. Thirdly, memories of the news events viewed the previous week were assessed in the same way (RN; real non-self news events).

Participants then performed a further three tasks in which they imagined novel plausible scenarios of three types. In each case they were instructed to imagine the event and describe it out loud with their eyes closed (previous pilot testing determined that eyes closed was most comfortable for the majority of subjects). Participants typically required 2–3 min to construct and describe out loud a rich and detailed imagined event based on each scenario. They were informed they would be required to recall the details of each imagined event in the future session. Poorly imagined or described events were excluded.

Participants were provided with cues concerning scenarios of common-place events that they had to imagine involving themselves (IS; imagined self events). They were instructed that the event should include active involvement of themselves (rather than a static scene), and to make it emotionally neutral in content. Importantly, participants were instructed to imagine a completely novel event independent of any previous specific event memories (e.g. not an actual past or potential future event). This was verified after each trial. Below is an example transcript of an imagined (autobiographical) self event from the experiment. The event is clearly dynamic, in a first person perspective, and in the present (rather than the past or future).

Cue: Imagine going for a walk by a stream in a forest

“I'm visiting some family and my auntie lets me borrow her car, a little red car, and I start to drive to the forest and I've found on my map there's a nice walk you can do by a stream that ends up at a waterfall. So I park the car and get out and start walking and it's a really nice day. Not much really happens on the way but you have to follow the stream and then you come to this beautiful waterfall and its not particularly big but there is a family there and there are a couple of kids playing in the water and so I sit there for a while...”.

Participants were then provided with scenario cues for plausible events that could have happened (but didn't) to the characters in the film “Sideways” (IF; imagined non-self film events). Participants were instructed to imagine the event as if it was playing out on a screen, with themselves absent from the scenario. Importantly, participants were instructed to create a novel event, independent of any specific personal memories or events from the film. This was verified after each scenario.

Participants were then provided with scenarios of plausible events that could be reported on a UK television news programme (IN; imagined non-self news events). Participants were required to imagine the event as if it was a typical news report (i.e. including reporter commentary, interviews and related video footage) playing out on a screen and with themselves absent from the scenario (e.g. they should not make themselves the reporter). Importantly, participants were instructed to create a novel event, independent of any personal event memories or any specific news event memories, and this was verified after each scenario.

In addition to performing these six event-based tasks, participants also performed two object-based tasks (viewing and imagining). Participants performed the two object tasks three times through the interview session, to ensure they were robustly encoded and were informed that they would be required to recall them in the future session. Firstly, participants viewed a series of single acontextual objects (accompanied by short text descriptions to aid encoding) presented successively on the monitor (RO; real objects) in a self-paced manner. Objects were easily identifiable common objects presented on a blank white background and participants were told not to think about possible associated contexts (verified regularly by the experimenter).

The experimenter then read short descriptions of everyday acontextual objects out loud to the participant (IO; imagined objects). Participants were required to construct a detailed image of each object in their mind's eye, and to imagine each object against a plain white background, devoid of associated background context (verified regularly by the experimenter). Participants were instructed to create a novel object based on the experimenter's description, rather than recalling a personally familiar object. Real objects were randomly allocated to three separate sets which were identical in nature. Similarly imagined objects were randomly allocated to three sets. Allocation of objects to the six conditions was counterbalanced across participants. The reason for having three sets of real and three sets of imagined objects was so that each of the six event conditions had its own baseline condition.

Stimuli were similar in terms of memory age, only differing by a few days. The recent real autobiographical memories elicited during the interview session in week 2 had a mean age of 14.9 days (SD 3.7 days) and were therefore formed an average of 21.9 days prior to the fMRI session. Memories for the events that occurred during the viewing session (RF and RN) were formed 14 days prior to the fMRI session, while those in the interview session (IS, IF, IN, RO, IO) were formed one week prior to the fMRI session.

#### MRI scan session (week 3)

One week after the interview session participants engaged in a memory recall task during fMRI scanning. Participants first performed a behavioural practice outside the scanner with stimuli not used in the main experiment. There were six event conditions: RS, RF, RN, IS, IF, IN, and six object control conditions: RO^1^, RO^2^, RO^3^, IO^1^, IO^2^, IO^3^ (see Table 1 for an overview of conditions). There were 120 trials in total: 60 event trials (10 trials per event condition); 30 real object trials (3 real object conditions × 10 trials) and 30 imagined object trials (3 imagined object conditions × 10 trials). Thus the number of trials in the event and object conditions was equated. There were 6 experimental runs each composed of 20 trials. Trial types were randomly intermixed through each run and across the whole experiment.

Each trial began with the presentation of a sentence text cue (6 s) spanning two lines that briefly described the event or object to recall (see Table 2 for example text cues). Following this, the text “Close your eyes now” was presented on the screen and participants had 20 s (determined after pilot testing) during which they internally (i.e. not out loud) recalled and re-experienced the cued event or object in as much detail as they could. They were instructed not to construct or elaborate novel details about the event/object. After 20 s an audio tone signalled the participant to open their eyes. They then performed three different ratings using an fMRI compatible 5-button keypad to provide an instant measure of their perceived performance on that trial. Participants rated difficulty: “How difficult did you find it to recall that memory?” (1 = very easy…5 = very difficult); vividness: “How vivid was your recalled memory?” (1 = not at all vivid…5 = very vivid) and perceived accuracy: “How accurately did you recall that memory?” (1 = not very accurately…5 = very accurately) with accuracy referring to how well they thought their recall matched the details of the previously experienced or imagined event/object when it was first described/created. Participants had 4 seconds to make each response. There was a 2 second delay before the next trial began.

#### Post-scan debriefing session

Immediately after the scanning session participants were thoroughly debriefed. The debriefing was digitally recorded for later verification purposes. Participants performed ratings (range 1 to 5 — see Results) for each condition. Two types of ratings of perspective/self-involvement were collected. For autobiographical events, which were instructed to be viewed from a 1st person perspective, participants were asked to rate whether they indeed recalled the event from this perspective, versus the alternative 3rd person perspective. This rating sought to ensure that not only were participants actively involved in the recalled autobiographical event, but that they recalled it from a 1st person perspective. For non-self events, participants were instructed to recall the event as if it was playing out in front of them, and not to be present in, or part of, the scenario. Consequently, we instead asked the participants to rate the degree of personal involvement or detachment. Although we did not directly ask them to rate 1st versus 3rd person perspective, we considered that this alternative rating assessed a more critical aspect of these events, ensuring that participants were not present and thus had to be recalling the event from an outsider/observer perspective. Participants also rated the dynamic or static nature of the event, the degree of emotional salience, the degree to which the participant associated a particular period of time with the event (“time stamp” e.g. past, future, none), and the plausibility of the event. The participant was also asked to describe out loud all the details recalled in the scanner for 2–3 example events from each condition, 10–15 real objects and 10–15 imagined objects. Descriptions were compared with details provided during the pre-scan interview session to confirm accurate recall.

### fMRI scanning parameters

T2⁎-weighted echo planar (EPI) images with blood oxygen level-dependent (BOLD) contrast were acquired on a 1.5 tesla Siemens AG (Erlangen, Germany) Sonata MRI scanner. Scanning parameters were selected to achieve whole brain coverage: 45 oblique axial slices angled at 30 degrees in the anterior–posterior axis, 2 mm thickness (1 mm gap), repetition time of 4.05 s. The first 6 ‘dummy’ volumes from each session were discarded to allow for T1 equilibration effects. A T1-weighted structural MRI scan was acquired for each participant after the functional scanning sessions.

### Data analysis

Data were analyzed using SPM5 (www.fil.ion.ucl.ac.uk/spm). Spatial preprocessing consisted of realignment and normalization to a standard EPI template in Montreal Neurological Institute (MNI) space with a resampled voxel size of 3 × 3 × 3 mm, and smoothing using a Gaussian kernel with full width at half maximum of 8 mm. Statistical analysis was performed using the general linear model. The experiment had 6 event conditions (RS, RF, RN, IS, IF, IN) which were compared against 6 object control conditions (RO^1^, RO^2^, RO^3^, IO^1^, IO^2^, IO^3^). The main time period of interest was the 20 second recall/re-experience interval while participants had their eyes closed. This period was modelled as a boxcar function (20 second duration) and convolved with the canonical haemodynamic response function to create regressors of interest. Participant-specific movement parameters were included in the design as regressors of no interest. Participant-specific parameter estimates relating to each regressor, collapsed across the 6 sessions, were calculated for each voxel. These parameter estimates were entered into a second level random-effects analysis using a one-way ANOVA. The full network of regions activated across all event types, minus their respective object baseline conditions, was investigated using the standard conjunction function implemented in SPM5 (global; *p* < 0.001 uncorrected; minimum cluster size of 5 voxels). Experimental conditions were then contrasted to further probe the functional contributions of regions within this network by entering the parameter estimates into a second level random-effect analysis using standard *t* test analyses. An additional parametric analysis was performed to investigate whether variations in the behavioural rating scores for difficulty, vividness and perceived accuracy modulated activity in any regions of the brain. All three behavioural ratings scores were entered as parametric modulators of interest for each condition type and their respective parameter estimates were entered into a second level random-effect analysis using standard *t* test analyses.

Regions of *a priori* interest for the autobiographical memory network, including cortical midline areas, were derived from previous studies and a meta-analysis (Addis et al., 2004, 2007; Buckner and Carroll, 2007; Cabeza and St Jacques, 2007; Gilboa et al., 2004; Hassabis et al., 2007a; Maguire, 2001a; Maguire and Frith, 2003a,b; Ryan et al., 2001; Schacter et al., 2007; Svoboda et al., 2006) and are specified in the first paragraph of the Introduction. For all contrasts we report areas in this entire network that survived using a threshold of *p* < 0.001 uncorrected for multiple comparisons with a minimum cluster size of 5 voxels. We employed a corrected threshold of *p* < 0.05 (FWE) for areas that were not hypothesised in advance, and none survived correction. Note that for complete transparency, in the tables we include all areas that survived using the threshold *p* < 0.001 uncorrected for multiple comparisons. However, we do not discuss areas on these tables that were not hypothesised in advance, and primarily focus our discussion on those areas of the memory network most germane to our main interest, namely cortical midline regions.

In addition, we also applied small volume correction. We used the main effect of all memory events minus the baseline object controls to establish the distributed network. We identified our regions of interest from this contrast (based on the extant literature alluded to previously). We noted the peak coordinate in each area at *p* < 0.001 (uncorrected). We used these coordinates to define the regions of interest (RoIs) for the contrasts summarised in Fig. 5 (where these contrasts relate to comparisons between the different event types). The RoIs were spheres of 8 mm around these peak coordinates. We then employed Bonferroni correction to take account of the number of regions in each hemisphere and adjusted the p values accordingly (left hemisphere, 6 regions, new corrected threshold *p* = 0.008; right hemisphere, 4 regions, new corrected threshold *p* = 0.01) to confirm these areas were significantly active at this corrected threshold. The peak coordinates from the main effect contrast used to define the RoIs were: right medial PFC (3,30,24), left medial PFC (− 6,− 30,− 12;− 3,54,27), left anterior cingulate cortex (− 3,18,− 9), left ventromedial PFC (− 9,42,− 12), left retrosplenial cortex (− 6,− 51,3;− 12,− 60,15), right posterior cingulate cortex (21,− 60,21), right precuneus (3,− 51,− 39;0,− 60,39). In addition, when considering activations in adjacent brain areas, only those whose peak voxels were more than the smoothing kernel (8 mm) apart were regarded as separate. All activations are displayed on sections of the average structural image of all the participants and conform to MNI coordinate space.

## Results

### Behavioural data

#### Interview session (week 2)

One week after viewing the film and news clips participants recalled on average 97.4% (SD 7.1) of film events and 96.0% (SD 5.6) of the news clips thus demonstrating robust memories for the non-self events.

#### MRI scan session (week 3)

During fMRI scanning participants rated three aspects of their performance during each trial: difficulty (1 = very easy...5 = very hard), vividness (1 = not at all vivid...5 = very vivid) and perceived accuracy of recall (1 = not very accurate...5 = very accurate). The three ratings were broadly comparable across the event and object conditions (see Table 3). Difficulty was rated low on average (mean 0.83, SD 0.34), while vividness (mean 3.9; SD 0.36) and perceived accuracy of recall (mean 3.7; SD 0.40) were rated high. Formal comparisons between the conditions were conducted using a repeated-measures ANOVA testing the factors of stimulus type (event; object), reality (real; previously imagined) and category (self; film; news). For all three behavioural measures (vividness, difficulty and perceived accuracy) event and object stimulus types were equivalent {all [*F*(1,17) < 1.8, *p* > 0.2]}. By contrast, significant differences were found across the factors of reality and category. Real events and real objects were recalled more easily, more vividly and with a greater degree of perceived accuracy than imagined events and imagined objects {all [*F*(1,17) > 33.5, *p* < 0.001]}, and this effect was observed for all three event categories (self, film and news). In addition, differences were observed between event categories. Self and film events were relatively well matched, only differing in the degree of perceived accuracy [*F*(1,17) = 5.5, *p* = 0.03], but not in terms of vividness or difficulty {both [*F*(1,17) < 4.1, *p* > 0.06]}. Both self and film events were recalled more easily, more vividly and with a greater degree of perceived accuracy than news events {all [*F*(1,17) > 20.5, *p* < 0.001]}.

#### Post-scan debriefing

A number of additional variables were probed in the post-scan debriefing session. The perspective taken and the degree of self-involvement were assessed for self and non-self events respectively. Autobiographical events were rated as being recalled more often from a 1st person perspective than from a 3rd person perspective (1 = 3rd person...5 = 1st person) for both real (mean 4.2, SD 1.3) and previously imagined (3.4, SD 1.8) events, as instructed. Interestingly, real self events were rated as being slightly more from a 1st person perspective than imagined self events (*t*(17) = 1.9, *p* = 0.04). For non-self events, participants were instructed to recall the events as if they were playing out in front of them, and to be personally detached and absent from the event. Participants rated this degree of self-involvement and, in accordance with this instruction, reported that they were personally detached (1 = detached...5 = involved) from both real and previously imagined non-self conditions [RF: 1.5 (SD 1.0); RN: 1.3 (SD 0.7); IF: 1.3 (SD 0.7) IN: 1.2 (SD 0.6)]. There was no significant difference between the ratings of detachment for non-self events {reality: [*F*(1,17) = 0.7, *p* = 0.4]; category: [*F*(1,17) = 1.7, *p* = 0.2]; interaction: [*F*(1,17) = 0.4, *p* = 0.8]}. In addition there were no significant differences in the plausibility ratings between imagined events, with all three categories rated as plausible {implausible = 1...plausible = 5; [IS: 3.7 (SD 1.2); IF: 3.8 (SD 1.2); IN: 3.2 (SD 1.2)]; category: [*F*(2,34) = 3.0, *p* = 0.6]}. Participants also rated the imagined self events as dynamic and with active involvement of themselves (1 = static...5 = dynamic), rather than simply a static scene (4.1, SD 1.2).

Ratings of the emotional salience of event conditions were also taken. Although all events were selected to be of neutral or slightly positive emotional salience, real events, especially real self events, were rated with higher emotional salience (1 = low...5 = high) than the other conditions {RS: 3.4 (SD 0.8); RF 2.4 (SD 0.9); RN 1.6 (SD 0.8); IS 2.3 (SD 1.0); IF 2.1 (SD 1.0); IN 1.3 (SD 0.5); reality × category interaction: [*F*(2,34) = 4.5, *p* = 0.02]}. Post hoc analyses showed that this reality × category interaction was primarily driven by real self events, rather than real events in general {Self > film: [*F*(1,17) = 7.1, *p* = 0.016]; Self > news: [*F*(1,17) = 6.8, *p* = 0.018]; Film > news: [*F*(1,17) = 0.0, *p* = 1.0]}.

In addition, participants also rated how much “in the past” they felt the recalled event was (1 = no time stamp...5 = in the past). As expected, real events, especially real self events, were typically rated as feeling more in the past [RS: 4.5 (SD 1.0); RF: 3.1 (SD 1.8); RN: 2.4 (SD 1.6)] than previously imagined events [IS: 1.5 (SD 1.0); IF: 1.6 (SD 1.2); IN: 1.9 (SD 1.5)], which were rated as having no time stamp {reality × category interaction: [*F*(2,34) = 12.9, *p* = 0.001]}. Post hoc analyses showed that, like emotional salience, this reality × category interaction was primarily driven by real self events {Self > film: [*F*(1,17) = 9.8, *p* = 0.006]; Self > news: [*F*(1,17) = 31.8, *p* < 0.001]; Film > news: [*F*(1,17) = 3.4, *p* = 0.083]}.

### Neuroimaging data

#### Overview

This rich data set is amenable to analysis in numerous ways. For the sake of clarity, here we report findings that speak directly to our main focus on cortical midline structures and how selfness and realness modulated activity in these regions. In all of the comparisons described below, each of the 6 event conditions (RS, RF, RN, IS, IF, IN), was first analyzed relative to its corresponding object control condition (RO^1^, RO^2^, RO^3^; IO^1^ IO^2^, IO^3^; 6 different sets). We included the behavioural ratings (difficulty, vividness, perceived accuracy) as covariates in a parametric analysis asking whether activity in any brain areas correlated with the scores. This analysis revealed no regions of *a priori* interest where activity significantly correlated with vividness, difficulty or perceived accuracy. This is most likely because the conditions were generally well matched and the scores were broadly similar.

#### Overall network

**Fig. 2:**
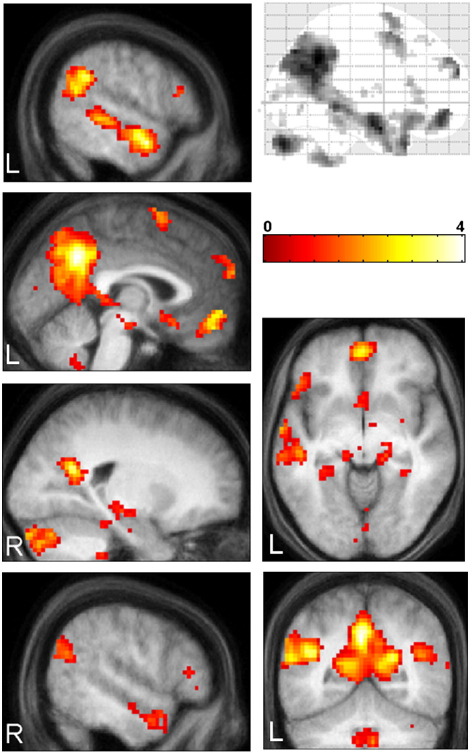
Brain areas activated in common for all event types. The brain areas engaged by all events types were revealed by a conjunction analysis. A widespread network of regions was activated including hippocampus, lateral temporal cortices, precuneus, posterior cingulate cortex, retrosplenial cortex, medial frontal regions and cerebellum. Table 4 details the coordinates of all activation peaks. Top right panel shows a sagittal image from a ‘glass brain’, which enables one to appreciate activations in all locations and levels in the brain simultaneously. The other panels show activations on a selection of relevant sagittal, coronal and axial sections from the averaged structural MRI scan of the 18 participants. R = right; L = left. The colour bar indicates the *Z*-score associated with each voxel.

We first sought to identify the distributed set of brain regions activated in common across the six event conditions. In this conjunction analysis we replicated the well-established network of brain areas reliably activated in studies of autobiographical memory, imagination of scenes, and episodic future thinking (Cabeza and St Jacques, 2007; Maguire, 2001a; Rugg et al., 2002; Svoboda et al., 2006), comprising the hippocampus, parahippocampal gyrus, lateral temporal cortex, RSC, PCC, precuneus, temporo-parietal junction, thalamus, cerebellum and mPFC (Fig. 2, Table 4). While many previous studies of autobiographical memory recall have focused on the role of the hippocampus (e.g. Addis et al., 2004; Gilboa et al., 2004; Maguire and Frith, 2003b; Ryan et al., 2001), hippocampal activity was not the primary interest of this study. Instead, activity in the hippocampus and underlying parahippocampal cortex was not apparent in any of the subsequent contrasts between event conditions, suggesting its activation reflects a common mechanism during retrieval, or perhaps a role in providing the spatial context for both real and imagined events (e.g. Hassabis et al., 2007a,b; Hassabis and Maguire, 2007; Addis et al., 2007; Schacter et al., 2007).

Having established the basic network involved in supporting events, we then focused on examining brain areas modulated by selfness and realness in order to functionally deconstruct the spatially extended midline frontal and posterior activations apparent in Fig. 2.

#### Regions modulated by real self and self

**Fig. 3:**
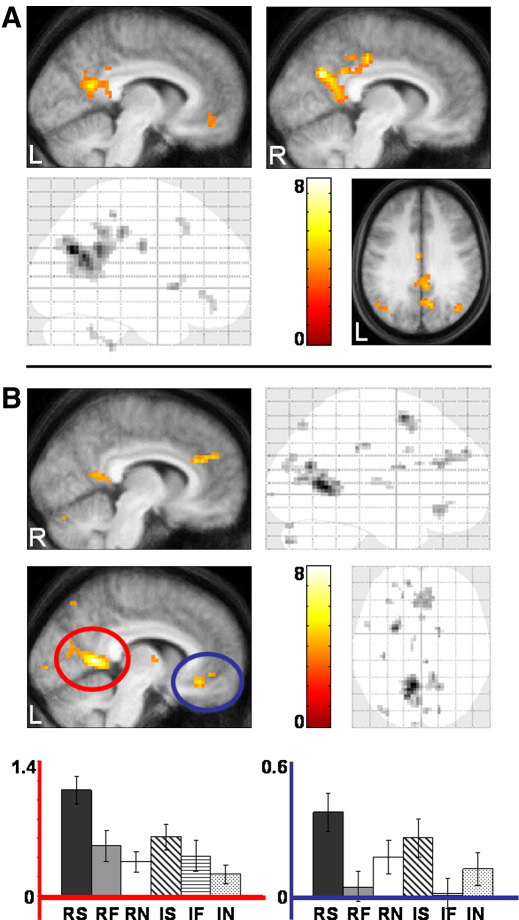
Brain areas preferentially engaged by the self. (A) Brain regions more active during the recall of real autobiographical memories (RS) compared to the recall of previously imagined autobiographical memories (IS). Table 5 details the coordinates of all activation peaks. Bottom left panel shows a sagittal image from a ‘glass brain’. The other panels show activations on a selection of relevant sagittal and axial sections from the averaged structural MRI scan of all participants. (B) Recalling autobiographical events (real and imagined: RS + IS) activated a distinct set of brain areas when compared with recalling non-self film and news events (real and imagined: RF + IF + RN + IN). Table 6 details the coordinates of all activation peaks. Right-sided panels show sagittal and axial images from a ‘glass brain’. The other panels show activations on two relevant sagittal sections from the averaged structural MRI scan of all participants. Below are the condition specific parameter estimates (betas) in arbitrary units at the peak voxels of two regions. Bars represent the standard error. Red = retrosplenial cortex, blue = ventromedial prefrontal cortex. R = right; L = left. The colour bars indicate the *Z*-score associated with each voxel.

As noted above, a first step in breaking down the functional subdivisions within this memory network was taken by Hassabis et al. (2007a). Here, unlike the study by Hassabis et al. (2007a), we were able to dissociate the factor of selfness from the factor of realness. When recall of real autobiographical (self) memories was compared with recalling self-relevant events that were previously imagined we found that several regions were more active during recall of real autobiographical events (real self > imagined self; Fig. 3A, Table 5). These included the PCC (especially on the right) extending into the precuneus, bilateral activations in the angular gyrus/temporo-parietal junction, and anteriorly in the mPFC specifically in ventromedial prefrontal cortex. Additional activations were observed in the frontal eye fields, and left and right cerebellum. The opposite contrast yielded no significant activations.

We were then able to go beyond previous studies by identifying brain regions that were driven specifically by the “self” aspect of an event (self > non-self; collapsed across real and imagined events). Several regions were more active when recalling self events compared to events of the other two non-self categories (film and news) (Fig. 3B, Table 6). These included activations posteriorly in the RSC extending dorsally along the parieto-occipital sulcus (POS; especially on the left), dorsal precuneus and in the left angular gyrus/temporo-parietal junction. Frontal activations were observed medially in the anterior cingulate cortex extending into the medial superior frontal gyrus, and in ventromedial prefrontal cortex. Laterally, there was increased activity in the left lateral orbital gyrus and frontal eye fields. Subcortical activations were apparent in the medial dorsal nuclei of the left thalamus and in the right head of the caudate nucleus.

**Fig. 4:**
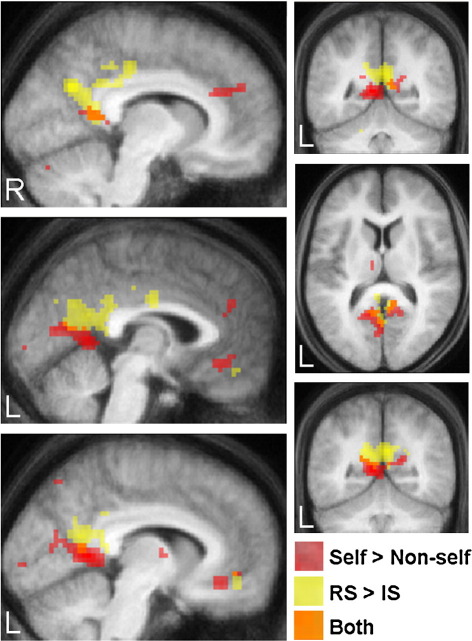
Comparison of brain regions modulated by self and real self. The brain regions preferentially activated when recalling real autobiographical (RS) events compared to imagined autobiographical (IS) events (yellow) are overlaid on the same brain (averaged structural MRI scan of all participants) as regions that were more active for recalling autobiographical events (real and imagined: RS + IS) compared to non-self (film and news, both real and imagined: RF + IF + RN + IN) events (red). Regions that were active in both contrasts are shown in orange. R = right, L = left.

In order to visualise the relationship between brain regions modulated by the “self” aspects of memory, Fig. 4 displays the results of the above contrasts (real self > imagined self; self > non-self) simultaneously. A striking distinction in the areas modulated for each contrast is apparent, with a small degree of overlap in some areas. The POS, dorsal anterior cingulate cortex and medial dorsal nucleus of the thalamus were preferentially involved in recalling self events (real or imagined) compared to non-self events. By contrast, the PCC, precuneus and right angular gyrus/temporo-parietal junction were preferentially activated for real autobiographical memories compared to previously imagined self events. In addition, there were two small regions where the activations overlapped, in RSC extending into the POS, and anteriorly in ventromedial prefrontal cortex (although see below).

#### Regions modulated by realness

This prominent distinction between regions that differentiated between self and non-self events, and regions which differentiated between whether that self event really happened or had only been imagined, provides novel insights into functional sub-regions within the vast swathes of midline cortical activity observed in Fig. 2. However, it is also clear from Fig. 2 that the sub-regions we have identified so far only encompass part of the large spatially extended midline activations. Using comparisons of our other experimental conditions we therefore sought to determine the functional roles of more areas across the cortical midline. We concentrated on areas within the range of + 12 to − 12 mm in the *x* direction as the majority of the relevant activations were focused across this region both in this and previous studies (e.g. Addis et al., 2007; Cabeza and St Jacques, 2007; D'Argembeau et al., 2008; Gilboa et al., 2004; Hassabis et al., 2007a; Maguire, 2001a; Maguire and Frith, 2003a; Maguire and Mummery, 1999; Schacter et al., 2007; Svoboda et al., 2006; Szpunar et al., 2007). To ensure that all regions survived correction at a more stringent threshold, small volume correction (SVC) was performed on the peak voxel within each region of interest across the cortical midline, with further correction for the number of regions under consideration.

**Fig. 5:**
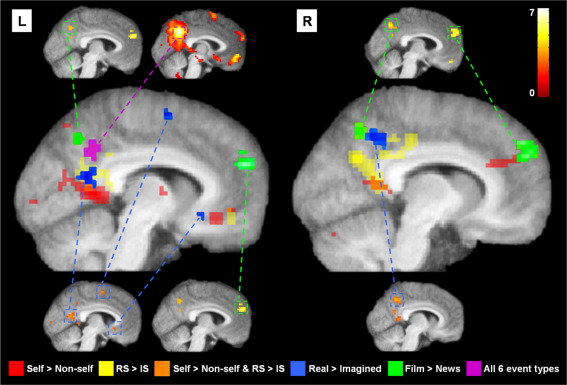
Functional subdivisions within cortical midline regions. The self-related overlays from Fig. 4′s medial sagittal sections are shown enlarged in the centre: in red are regions more active for recalling self events compared to non-self events, in yellow are areas engaged more for recalling real self (RS) events compared to imagined self (IS) events, in orange is the overlap between the two (see legend of Fig. 4 and Results). Midline cortical activations observed when recalling real events compared to imagined events (all categories together) are overlaid in blue, with the results of the actual SPM contrast shown in smaller sagittal sections on the periphery. Midline cortical activations observed when recalling film events compared to news events (collapsed across real and imagined events) are overlaid in green, with the result of the actual SPM contrast shown in smaller sagittal sections on the periphery. Overlaid in magenta is the peak region of the precuneus active during all six event conditions, with the actual SPM of the conjunction analysis also shown in a smaller sagittal section on the periphery. See text and tables for details of coordinates. R = right, L = left. The colour bar in the top right indicates the *Z*-score associated with each voxel for the smaller sagittal sections located on the periphery. The colour code along the bottom indicates each contrast displayed on the enlarged brains in the centre.

Above, we identified regions that were more active when recalling real compared to previously imagined autobiographical/self events. To explore this further, we extended the comparison to include all category types together (self, film and news). This allowed us to determine the cortical midline areas that were more active when recalling real events of all types compared to previously imagined events (real > imagined; collapsed across all 3 event categories). We found real events were associated with activity in the left PCC, right precuneus, and anteriorly in the pre-supplementary motor area (pre-SMA) and ventral anterior cingulate cortex. Fig. 5 shows these midline activations (in blue, see also Table 7) relative to the regions activated by the two self contrasts (self > non-self and RS > IS) shown in Fig. 4. All regions shown in Fig. 5 survived SVC except a small activation in the ventromedial PFC (yellow) in the real self > imagined self condition. Activations are also shown relative to the peak activation from the central portion of the precuneus (see Fig. 2, Table 4) which was active for all 6 event conditions (shown in magenta on Fig. 5). As might be expected, activity associated with recalling real events of all categories in left PCC partially overlapped with activations observed in the real self > imagined self contrast. The right precuneus activation was dorsal to regions in right PCC/precuneus activated for real self events. An additional activation was observed bilaterally in the RSC for real events. This coincided with areas that showed overlap in Fig. 4. However, the parameter estimates (betas) revealed that the activation was overwhelmingly driven the real self condition rather than by a main effect of realness (RS: 0.9; RF: 0.2; RN: 0.2; IS: 0.3; IF: 0.06; IN: 0.004). Anteriorly, the two (dorsal and ventral) medial frontal activations for real events were distinct from the anterior region modulated by self. The opposite contrast, comparing previously imagined events with real events, revealed no significant differences in activity across midline brain regions.

#### Further deconstruction of midline cortical regions

The inclusion of three different categories of events (self, film and news) allowed us not only to explore the neural correlates of self but also to make comparisons between two types of non-self events. Non-self events were selected from a feature length film or from news events seen on TV. Comparisons between film events and news events (collapsed across real and imagined events) revealed differences in a number of midline regions that were more active when recalling film than news events. These included the dorsal precuneus, and anteriorly the medial superior frontal gyrus (green areas on Fig. 5; see also Table 8). Interestingly, the superior frontal region was also more active for self events (real and imagined conditions together) compared to news events (3,51,27; *Z*-score = 4.14). The opposite contrast examining brain regions that were more active for recalling news events compared to film events (or self events) yielded no significant differences across midline brain regions.

## Discussion

Two defining features of autobiographical experiences are their relevance to the self and their occurrence in the real world. Despite the importance of understanding how one's personal experience of an event is represented, and the obvious advantage of being able to distinguish an event which really happened from one which was simply created internally, the neural correlates of selfness and realness remain unclear in the context of autobiographical memory. In the current study we experimentally manipulated these factors using a novel paradigm that enabled us to identify brain areas specifically modulated by the self-relevance of experiences, and to dissociate these from areas engaged by the realness of events. Therefore in this experiment we were able to effect a functional deconstruction of anterior and posterior cortical midline regions that are widely associated with autobiographical memory during neuroimaging (Cabeza and St Jacques, 2007; Maguire, 2001a; Svoboda et al., 2006). All recalled events were recent and well matched for memory age. Nevertheless, our task design dictated minor differences in age between real and imagined conditions (RS versus IS: average 14 days; RF/RN versus IF/IN: 7 days) and between self and non-self conditions (average 7 days). However, given that all events were very recent, and activity in the hippocampus, the primary region shown to be modulated by memory age (e.g. Gilboa et al., 2004; Maguire and Frith, 2003b; Ryan et al., 2001), did not differ across our event conditions, it is unlikely that memory age significantly affected our results.

### Anterior midline regions

While sub-divisions within medial prefrontal cortex have been explored and debated in various domains of cognition (e.g. Amodio and Frith, 2006; Gilbert et al., 2006; Northoff and Bermpohl, 2004; Ongur et al., 2003; Ramnani and Owen, 2004; Rushworth et al., 2007a,b), there has been a paucity of such work in the context of autobiographical memory. Here we identified distinct dorsomedial and ventromedial prefrontal areas that were modulated by selfness and realness. Recalling self events compared to non-self events activated a region in the right dorsomedial prefrontal cortex extending from the anterior cingulate cortex into the superior frontal gyrus. Interestingly, this area was not observed in the full common network (Fig. 2) suggesting it makes a specific contribution to autobiographical events. In support of this, a recent study investigating real autobiographical memory recall compared to an imagined non-self baseline task observed activation in this region (Szpunar et al., 2007), and it has been engaged during other self-representation studies (e.g. D'Argembeau et al., 2007; Sugiura et al., 2005), suggesting this area is associated with self-referential processing during memory recall. This anterior cingulate region was flanked anteriorly by a region in the superior frontal gyrus which was active in all conditions but showed greater enhancement for both the self and film events (real and imagined) compared to the news condition. This region is commonly activated in studies of mentalizing about self or others, (Blakemore and Decety, 2001; Gallagher and Frith, 2003; Gallagher et al., 2000; Johnson et al., 2002; Mitchell et al., 2005a,b; Ochsner et al., 2004; Olsson and Ochsner, 2008). In the current experiment, real and imagined self and film events share similarities in their naturalistic rich character knowledge, and their recall required participants to reflect on the mental states, thoughts and actions of themselves, and the film characters. In contrast, news events had sparse character knowledge, limiting the likelihood of self/other mentalizing. Thus these data support the view that this superior frontal region mediates mentalizing processes about the self and other individuals.

There was one additional dorsal region located within the pre-SMA where activity was enhanced when participants recalled real events of all categories compared to imagined events. Activity within this region has commonly been observed in studies of cognitive control processes such as in conflict, uncertainty and error monitoring (Amodio and Frith, 2006; Holroyd et al., 2004; Ridderinkhof et al., 2004; Rushworth et al., 2007b), as well as in studies of autobiographical memory (Gilboa et al., 2004; Hassabis et al., 2007a) and self-relevance judgements (D'Argembeau et al., 2007; Johnson et al., 2002; Mitchell et al., 2006; Moran et al., 2006). It has been suggested this region may mediate performance monitoring processes such as the interplay between internal and external demands (Gusnard et al., 2001; Holroyd et al., 2004; Ridderinkhof et al., 2004). One possible reason for activation of this region in the current study could be that monitoring recall performance relative to experienced real events may have up-regulated this region compared with events that were only ever experienced internally.

Selfness and realness also activated adjacent areas in left ventromedial prefrontal cortex. The most posterior activation was in ventral anterior cingulate (subgenual) cortex and was engaged when recalling real events of all categories compared to imagined events. A number of recent studies have suggested this region may mediate the interaction between the emotional significance and the self-relevance of stimuli (Moran et al., 2006; Sharot et al., 2007; van den Bos et al., 2007). It has also been activated in studies of reality monitoring using simplified stimuli (Simons et al., 2008; Turner et al., 2008). A more anterior region located within the orbitomedial prefrontal cortex was activated by self more than non-self events. This was flanked anteriorly by a region more active when processing real self events compared to imagined self events, although this latter region did not survive small volume correction. Previous studies of self-representation, confabulation, and autobiographical memory recall have implicated the ventral portion of the mPFC, especially when the stimuli have explicit emotional associations (Gusnard et al., 2001; Kjaer et al., 2002; Northoff et al., 2007; Piefke et al., 2003; Schnider, 2003; van den Bos et al., 2007). In general the ventral/orbitomedial prefrontal cortex has been engaged by tasks involving emotion and reward behaviours (e.g. Bechara et al., 2000; Breiter et al., 2001; Knutson et al., 2001; O'Doherty et al., 2003; Rolls, 2000; van den Bos et al., 2007). Although stimuli in the current study were selected so their emotional valence was neutral, subjective emotional ratings showed that real events and in particular real self events were more emotionally salient. This suggests that real events have a greater emotional impact during recall perhaps due to the manner in which they were initially experienced and perceived in an external multi-sensory context. By contrast, internally generated events only occurred within the imagination and their salience may be less, despite similar amounts of subjective emotional descriptors in the event content.

### Posterior midline regions

Posteriorly the PCC, precuneus, and RSC, have been consistently engaged during studies of autobiographical memory recall, self-representation, and future thinking (Addis et al., 2007; D'Argembeau et al., 2007; Daselaar et al., 2008; Gilboa et al., 2004; Hassabis et al., 2007a; Johnson et al., 2002; Maguire, 2001a; Northoff et al., 2006; Schacter et al., 2007; Sugiura et al., 2005; Svoboda et al., 2006; Szpunar et al., 2007; Vogeley and Fink, 2003). Despite the swathes of activity that encompass this large spatially-extended region during these tasks, anatomical and connectivity patterns point to distinct underlying sub-regions (Cavanna and Trimble, 2006; Vogt and Laureys, 2005). In this study, the central portion of the precuneus was prominently active across all six event conditions (see Fig. 2). This may reflect a common imagery mechanism involved in the recall of all types of complex events. In support of this, several studies have implicated the precuneus in imagery and visualisation of visual-spatial information in perception and memory (Cavanna and Trimble, 2006; Fletcher et al., 1995). Interestingly, we identified functional sub-divisions within the precuneus. A more dorsal region (on the right) was preferentially engaged for real events compared to imagined events across all categories. Posterior to this, an area of precuneus was more active for film events compared to news events. Increased requirements for imagery and visualisation inherent in real events, and in film more than news events, may have recruited additional precuneus resources.

Moving ventrally into PCC, we observed activity in right PCC extending dorsally into the precuneus and ventrally into the POS in response to real autobiographical events. Within PCC itself, however, we noted possible lateralised sub-divisions. As well as right PCC being modulated by real self events, parts of the left PCC were more active for real events compared to imagined events across all categories, reflecting a more general modulation by the experiencing of real external events, and not specific to self-processing. The PCC/precuneus has been implicated in many studies involving memory retrieval and familiarity (e.g. Hornberger et al., 2006; Rugg et al., 2002; Vincent et al., 2006; Wagner et al., 2005) and self representation (Addis et al., 2007; Hassabis et al., 2007a; Johnson et al., 2002; Lou et al., 2004; Moran et al., 2006; Ochsner et al., 2004; Sugiura et al., 2005; Vogeley et al., 2004; Vogt et al., 2006). In a previous study, Hassabis et al. (2007a) showed that recalling familiar events compared to newly imagining events activated portions of PCC/precuneus. In addition, Sugiura et al. (2005) found that parts of PCC were involved in representing the familiarity of a stimulus, while a distinct PCC sub-region coded specifically for personally familiar places. Similarly, our findings support the idea that portions of PCC/precuneus distinguish between the more familiar real events, compared to imagined events. Furthermore, other areas of PCC (especially on the right), which overlap with the PCC region specific for personally familiar events described by Sugiura et al., are particularly involved in representing real personal events. Thus our results add further weight to the view that during autobiographical memory retrieval the cooperation between self-processing and familiarity functions performed by these distinct subregions of the PCC/precuneus, along with the mPFC, may enable the brain to distinguish between real and imagined experiences (Hassabis et al., 2007a; Hassabis and Maguire, 2007).

By contrast, activity along the ventral portion of the left POS, extending into the left and right RSC, coded for the self perspective in both real and imagined self events. Interestingly, this POS/RSC activation was more lateralised to the left hemisphere. The function of the RSC, tucked behind the posterior bend of the corpus callosum, has proved difficult to establish, with previous studies suggesting it is involved in both autobiographical memory retrieval and topographical and visual–spatial processing (Bar and Aminoff, 2003; Byrne et al., 2007; Epstein and Higgins, 2007; Maguire, 2001a,b; Svoboda et al., 2006; Wolbers and Buchel, 2005). Indeed, the same POS/RSC region was observed in another study of autobiographical memory recall when a non-self condition was used as a control task (Szpunar et al., 2007), as well as in other studies of autobiographical memory recall as part of larger swathes of activation (Addis et al., 2007; Hassabis et al., 2007a; Maguire and Frith, 2003b; Maguire and Mummery, 1999). Recent studies have postulated that the ventral POS and RSC may mediate the transformation between self-centred and world-centred spatial reference frames or viewpoints during memory recall, imagery and navigation (Burgess, 2006; Byrne et al., 2007; Ino et al., 2002). Its anatomical position and connectivity with posterior parietal and medial temporal lobe regions make it ideal for such a role (Kobayashi and Amaral, 2003, 2007). In the current study, recalling self events required the participants to constantly update and transform their personal perspective relative to the spatial context of the environment, as they progressed through the unfolding event. By contrast, the detached viewpoint from which participants recalled the non-self events, which were similarly dynamic in nature but with no self-involvement, required less self-referencing relative to the environmental context as the event unfolded. The left POS/RSC, possibly in association with other regions such as the frontal eye fields, which were also more active during retrieval of self events (see Tables 5 and 6; see also Wallentin et al., 2008), and hippocampus, may interact with the mPFC and PCC to provide a functional framework mediating self-referencing and self-relevance during reconstruction of autobiographical events.

Overall, we have shown that regions within posteriomedial cortex and mPFC play distinct roles in processing key aspects of autobiographical memories. We have moved from the gross activations typically reported in autobiographical memory studies visible in Fig. 2, to a more refined parcellation shown in Fig. 5. Identification of these sub-divisions is an important step in functionally deconstructing the cortical midline, with implications for understanding not just the mechanisms underpinning autobiographical memory, but also other crucial cognitive operations such as thinking about the future and navigation (Buckner and Carroll, 2007; Hassabis and Maguire, 2007). In the future it will be important to establish how these areas interact with each other and with other parts of the memory network such as the medial temporal lobe to support the recollective experience.

## Figures and Tables

**Table 1:** Summary of experimental conditions

Abbreviation	Description
*Event conditions*	
RS	Recall of recent autobiographical (self) memories elicited in the pre-scan interview
RF	Recall of film event memories elicited in the pre-scan interview
RN	Recall of news event memories elicited in the pre-scan interview
IS	Recall of imagined autobiographical (self) events previously constructed in the pre-scan interview
IF	Recall of imagined film events previously constructed in the pre-scan interview
IN	Recall of imagined news events previously constructed in the pre-scan interview

*Object control conditions*	
RO^1^	Recall of acontextual objects visually presented in the pre-scan interview (set 1)
RO^2^	Recall of acontextual objects visually presented in the pre-scan interview (set 2)
RO^3^	Recall of acontextual objects visually presented in the pre-scan interview (set 3)
IO^1^	Recall of imagined acontextual objects previously constructed in the pre-scan interview (set 1)
IO^2^	Recall of imagined acontextual objects previously constructed in the pre-scan interview (set 2)
IO^3^	Recall of imagined acontextual objects previously constructed in the pre-scan interview (set 3)

**Table 2:** Examples of text cues used in the memory recall task during scanning

*Events*	
RS	Recall when you went to see the Japanese play in Notting Hill with friends
RF	Recall when Miles and Jack stopped at Miles' mother's house for dinner
RN	Recall when the man in Dublin invented the new sideways bicycle
IS	Recall imagining when you visited a bustling street market one morning
IF	Recall imagining when Miles went to a bookshop in town for a literary event
IN	Recall imagining when parents with quads met in San Diego for the convention

*Objects*	
RO	Recall the pink and orange suede wallet with a leather-effect front pocket
IO	Recall imagining the red china teapot with a busy oriental silver leaf design

**Table 3:** Behavioural ratings from the memory recall task during scanning

Conditions	Ratings (1 = low … 5 = high): mean (SD)		
	Difficulty	Vividness	Accuracy
Previously experienced autobiographical events (RS)	0.29 (0.37)	4.47 (0.43)	4.39 (0.45)
Previously viewed film events (RF)	0.34 (0.25)	4.34 (0.46)	4.20 (0.49)
Previously viewed news events (RN)	0.82 (0.51)	3.80 (0.66)	3.63 (0.60)
Previously imagined autobiographical events (IS)	0.89 (0.61)	3.85 (0.69)	3.66 (0.65)
Previously imagined film events (IF)	1.20 (0.44)	3.60 (0.60)	3.33 (0.64)
Previously imagined news events (IN)	1.59 (0.63)	3.13 (0.77)	2.86 (0.78)
Previously viewed objects (RO)	0.72 (0.46)	4.12 (0.48)	3.93 (0.56)
Previously imagined objects (IO)	0.91 (0.57)	3.79 (0.56)	3.68 (0.62)

**Table 4:** Brain regions activated by all event types (conjunction analysis)⁎

Region	Peak coordinate (*x*, *y*, *z*)			*Z*
Left medial superior frontal gyrus	− 9	63	24	> 8
Medial ventral frontal cortex	0	48	− 12	> 8
Left anterior cingulate cortex (ventral)	− 3	18	− 15	5.05
Left medial pre-supplementary motor area	− 3	6	69	6.21
Left superior frontal gyrus	− 21	39	48	3.82
Left middle frontal gyrus–frontal eye fields	− 36	6	57	6.91
Left inferior frontal gyrus	− 54	21	15	5.04
Left inferior frontal gyrus	− 51	30	0	4.81
Right inferior frontal gyrus	57	30	9	4.81
Left thalamus	− 15	− 15	9	4.14
Right superior temporal gyrus	45	15	− 33	6.83
Left superior temporal sulcus	− 57	− 3	− 21	> 8
	− 51	− 42	− 3	> 8
Right superior temporal sulcus	48	− 36	− 3	3.76
Right hippocampus	18	− 30	− 9	4.98
Left parahippocampal gyrus	− 24	− 36	− 15	5.61
Right parahippocampal gyrus	30	− 36	− 18	4.90
Left precuneus	− 3	− 54	36	> 8
Left posterior cingulate	− 6	− 42	39	> 8
Right posterior cingulate	12	− 54	30	5.87
Left retrosplenial cortex	− 9	− 54	3	6.36
Left parietal-occipital sulcus	− 12	− 60	12	7.76
Right parietal-occipital sulcus	21	− 60	21	> 8
Left angular gyrus/temporo-parietal junction	− 48	− 69	30	> 8
Right angular gyrus/temporo-parietal junction	42	− 63	27	6.69
Occipital cortex	0	− 69	− 3	4.40
	6	− 87	− 18	4.33
Right cerebellum	9	− 48	− 48	6.71
Left posterior cerebellum	− 27	− 81	− 36	4.94
Right posterior cerebellum	27	− 84	− 33	> 8

⁎ The contrasts entered into the conjunction analysis were: (RS–RO^1^) (RF–RO^2^) (RN–RO^3^) (IS–IO^1^) (IF–IO^2^) (IN–IO^3^).

**Table 5:** Recall of real autobiographical events > recall of imagined autobiographical events⁎

Region	Peak coordinate (*x*, *y*, *z*)			*Z*
Left medial ventral frontal cortex	− 9	42	− 9	3.45
Right anterior cingulate cortex	3	33	− 3	3.49
Left middle frontal gyrus–frontal eye fields	− 21	24	45	3.49
Right insula	39	12	0	3.97
Right postcentral gyrus	30	− 33	54	3.84
Left posterior cingulate cortex	− 3	− 12	30	4.08
Right parietal occipital sulcus	12	− 66	27	5.32
Left angular gyrus/temporo-parietal junction	− 48	− 66	33	3.54
Right angular gyrus/temporo-parietal junction	42	− 72	36	4.05
Left cerebellum	− 21	− 36	− 42	3.93
	− 18	− 51	− 30	3.57
Right cerebellum	27	− 51	− 33	3.44

⁎ (RS–RO^1^) > (IS–IO^1^).

**Table 6:** Recall of autobiographical events (real + imagined) > recall of film and news events (real + imagined)⁎

Region	Peak coordinate (*x*, *y*, *z*)			*Z*
Left lateral superior frontal gyrus	− 12	54	33	3.97
	− 27	51	27	3.45
	− 18	39	33	3.71
Left lateral orbital frontal gyrus	− 33	36	− 12	3.70
Left medial ventral frontal cortex	− 6	33	− 12	3.81
Right anterior cingulate gyrus	6	33	24	3.88
	3	18	24	3.55
Left middle frontal gyrus–frontal eye fields	− 24	6	60	4.56
Left inferior frontal gyrus	− 51	9	33	3.45
Left postcentral gyrus	− 36	− 24	39	3.81
Right caudate nucleus	18	6	21	3.91
Left thalamus	− 9	− 15	12	3.41
Right posterior cingulate cortex	6	− 30	36	3.86
Left retrosplenial cortex	− 6	− 54	6	5.08
Left precuneus	− 9	− 72	54	3.66
Left angular gyrus/temporo-parietal junction	− 39	− 81	27	3.58
Left calcarine sulcus	− 6	− 96	0	3.56
Right posterior cerebellum	18	− 84	− 36	3.85

⁎ [(RS + IS) − (RO1 + IO1)] > [(RF + IF + RN + IN) − (RO2 + IO2 + RO3 + IO3)].

**Table 7:** Recall of previously viewed (‘real’) events > recall of previously imagined events⁎

Region	Peak coordinate (*x*, *y*, *z*)			*Z*
Left medial pre-supplementary motor area	− 3	− 3	63	3.96
Left anterior cingulate cortex (ventral)	− 3	18	− 6	3.73
Right postcentral gyrus	48	− 9	54	3.69
Right superior temporal sulcus	60	3	− 18	3.78
Left retrosplenial cortex	− 9	− 45	0	3.23
Right retrosplenial cortex	9	− 51	6	5.67
Right precuneus	3	− 51	45	4.12
Left posterior cingulate cortex	− 15	− 57	18	4.36
Left calcarine sulcus	− 9	− 96	6	3.38

⁎ [(RS + RF + RN) − (RO^1^ + RO^2^ + RO^3^)] > [(IS + IF + IN) − (IO^1^ + IO^2^ + IO^3^)].

**Table 8:** Recall of film events (real + imagined) > recall of news events (real + imagined)⁎

Region	Peak coordinate (*x*, *y*, *z*)			*Z*
Medial superior frontal gyrus	0	54	27	4.55
Left medial ventral frontal cortex	3	45	− 21	3.72
Right middle temporal gyrus	48	12	− 33	4.57
	60	− 9	− 18	3.93
Right superior temporal sulcus	48	− 33	− 6	4.80
Left angular gyrus/temporo-parietal junction	− 57	− 60	30	3.89
Right angular gyrus/temporo-parietal junction	57	− 57	24	3.82
Precuneus	0	− 60	42	3.99

⁎ [(RF + IF) − (RO^2^ + IO^2^)] > [(RN + IN) − (RO^3^ + IO^3^)].
